# The dangers of “Chasing the dragon”: a fatal case of heroin-induced leukoencephalopathy

**DOI:** 10.1093/bjrcr/uaad004

**Published:** 2023-12-13

**Authors:** Iliass Bourekba, Ismail Halfi, Najwa Ech Cherif Kettani, Meryem Fikri, Mohamed Jidane, Firdaous Touarsa

**Affiliations:** Department of Neuroradiology, “Hôpital des Spécialités”, Rabat, 10170, Morocco; Department of Neuroradiology, “Hôpital des Spécialités”, Rabat, 10170, Morocco; Department of Neuroradiology, “Hôpital des Spécialités”, Rabat, 10170, Morocco; Department of Neuroradiology, “Hôpital des Spécialités”, Rabat, 10170, Morocco; Department of Neuroradiology, “Hôpital des Spécialités”, Rabat, 10170, Morocco; Department of Neuroradiology, “Hôpital des Spécialités”, Rabat, 10170, Morocco

**Keywords:** heroin-induced leukoencephalopathy, toxic encephalopathy, spongiform encephalopathy

## Abstract

Heroin-induced leukoencephalopathy (HLE) is a rare toxic encephalopathy associated primarily with heroin inhalation, commonly referred to as “chasing the dragon.” This study presents a clinical case of a 27-year-old polydrug user diagnosed with HLE during hospitalization for rapidly progressive flaccid tetraplegia and aphasia. The clinical manifestations encompassed cerebellar and bulbar dysfunction, coupled with motor impairment and altered consciousness. Based on the clinical data and MRI results, HLE was identified as the most likely cause. This article aims to provide insights into the clinical and radiological aspects of HLE, emphasizing the diagnostic significance of radiological findings. The gold standard examination for diagnosis is MRI, crucial due to the difficulties in obtaining histological confirmation for this rare condition.

## Introduction

Heroin-induced leukoencephalopathy (HLE) is a rare form of toxic encephalopathy that involves damage to the white matter, particularly the myelin, caused by heroin consumption. This condition is almost exclusively encountered when the patient inhale fumes resulting from heating the drug on tin-foil. It was first described in 1982 in the Netherlands, in 47 patients who presented with a “spongiform” leukoencephalopathy. The exact cause and pathophysiology of these lesions are still poorly understood to this day. Several hypotheses have been proposed, but the exact pathogenic agent remains unidentified.^1^^,^^2^

The clinical presentation typically includes signs of cerebellar and bulbar dysfunction that may be associated with motor impairment and altered consciousness. We present the case of a 27-year-old polydrug user in whom HLE was diagnosed following hospitalization for a rapidly progressive flaccid tetraplegia and aphasia. This article aims to present a typical case from both clinical and radiological perspectives, and to highlight the radiological findings that contribute to the diagnosis.

## Case report

A 27-year-old male patient, with a history of polydrug use, was admitted to our hospital for management of ataxia, rapidly progressive flaccid paraplegia and aphasia. Obtaining information from the patient and family was challenging, there was a history of chronic toxic substance abuse, including solvents such as toluene and n-hexane; however, heroin use was not initially reported. The symptoms started 12 days earlier with progressive muscle weakness and gait ataxia that evolved into paraplegia and aphasia, the communication was only possible through eye and nodding movements. Clinical examination revealed a conscious patient (GCS 15/15) with flaccid paraplegia and akinetic mutism. No fever or sensory impairment was noted.

**Figure 1.**
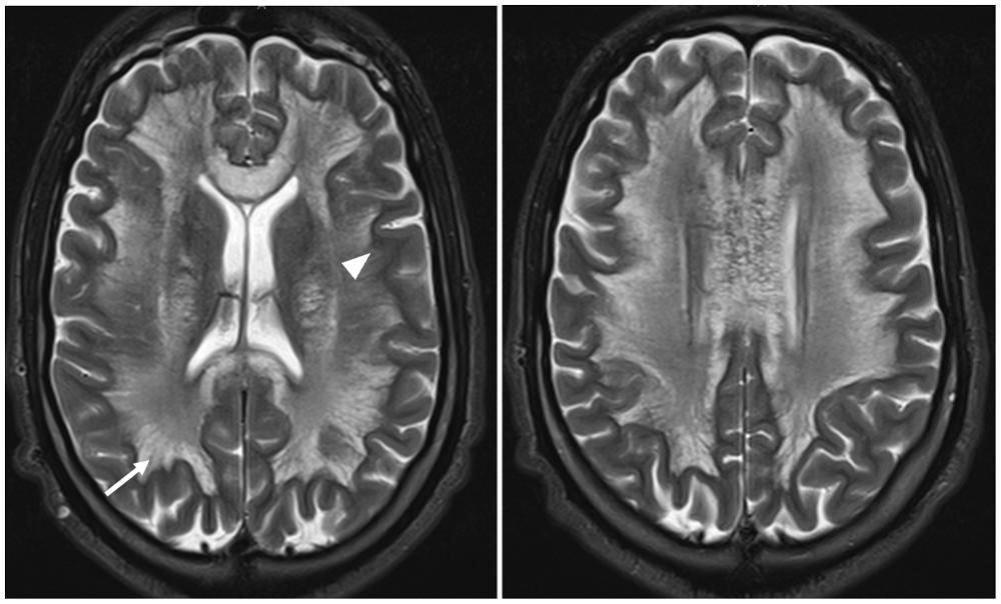
Axial T2-weighted slices depict diffuse bilateral white matter lesions respecting the U fibres (arrowhead) and exhibiting the characteristic “spongiform” appearance described in HLE, this refers to the visualization of vacuoles with a quasi-liquid signal located between the nerve fibres (arrow). Abbreviation: HLE = heroin-induced leukoencephalopathy.

**Figure 2.**
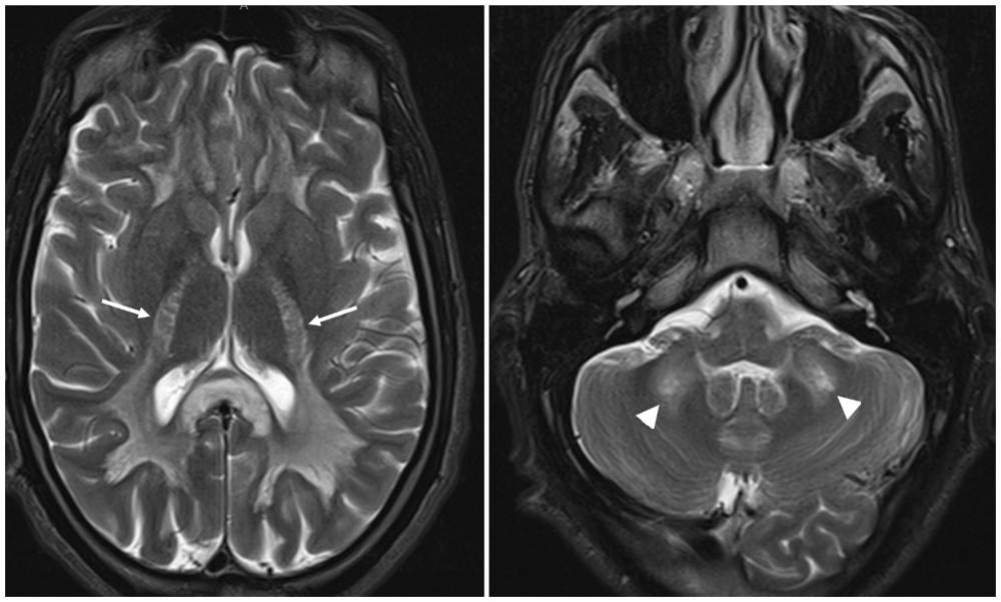
Axial T2-weighted slices passing through the thalamus and the posterior cranial fossa, demonstrate involvement of the posterior limb of the internal capsule (arrow) and the middle cerebellar peduncle sign or MCP sign (arrowhead).

**Figure 3.**
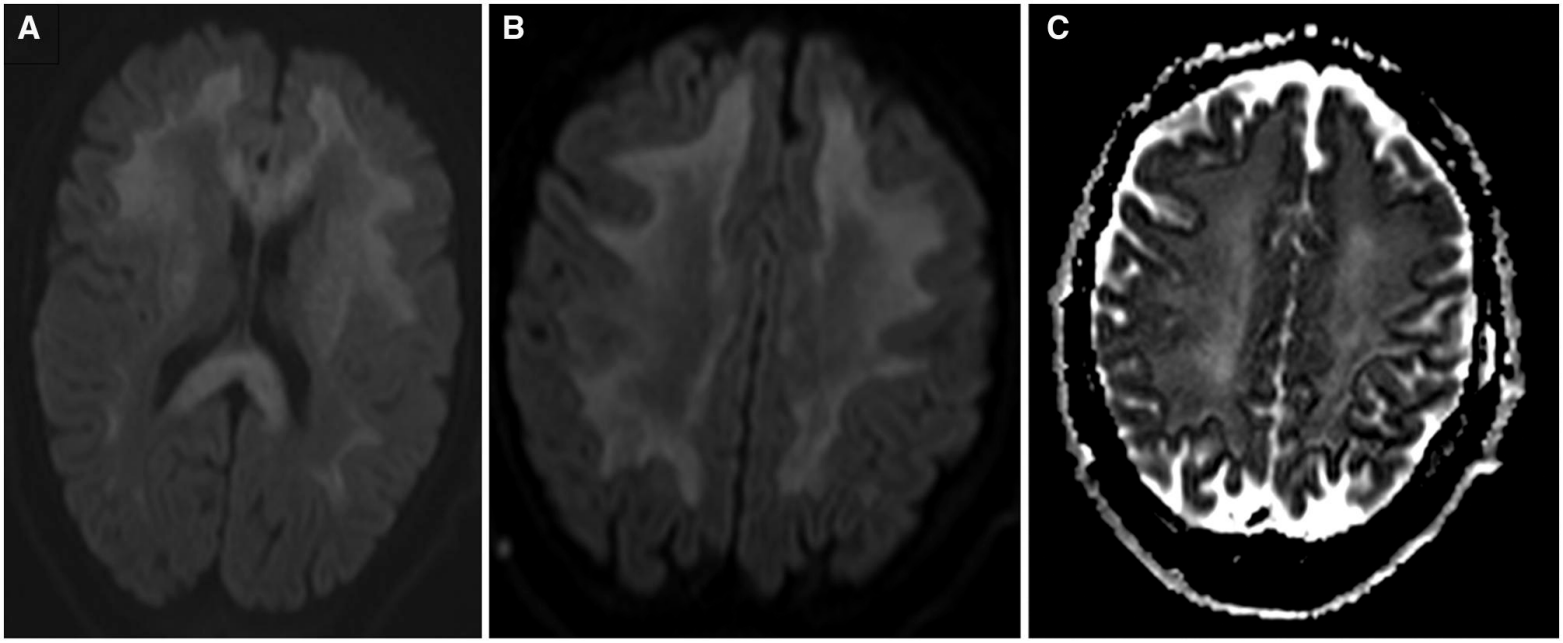
DWI images at two different levels illustrating diffusion restriction scattered within the white matter lesions (A, B), confirmed on the ADC cartography (C). Abbreviation: DWI = diffusion-weighted imaging.

MRI was performed to rule out an infectious disease within the central nervous system and potentially confirm the diagnosis of toxic encephalopathy. It revealed bilateral hemispheric signal abnormalities, hypointense on the T1-weighted sequence, and hyperintense on T2-weighted and FLAIR images (Figure 1). These lesions spared subcortical U-fibres and presented a “vacuolated” appearance, particularly visible on the T2-weighted sequence. Involvement of the posterior limb of the internal capsule extending to the corticospinal tract of the pons and symmetric involvement of the middle cerebellar peduncles (MCP sign) was also noted (Figure 2). The lesions also showed peripheral restrictive areas observed on the diffusion sequence and apparent diffusion coefficient (ADC) cartography (Figure 3). No pathological enhancement or signs of intracranial infection were observed. MR spectroscopy was not performed for this patient.

Lab tests including HIV serology, toxoplasmosis, and Genexpert^®^, came back negative. A blood and urine toxicology screening could only be conducted 11 days after hospitalization, it detected traces of benzodiazepines and tetrahydrocannabinol. However, a thorough re-interrogation revealed that the patient had used other types of drugs, including heroin which was consumed in its inhaled form 28 days earlier (which means a 16-day interval between drug consumption and the onset of symptoms). The absence of heroin detection in the toxicology tests is most likely explained by the delayed timing of the test.

Upon admission, the patient was initially placed on empirical antibiotic therapy which was later discontinued after encephalitis was ruled out. Over the next few days following the diagnosis, the patient received symptomatic treatment aimed at alleviating his symptoms; however, his condition continued to worsen. On day 22 after his hospitalization, he experienced status epilepticus and remained in an altered state of consciousness thereafter. He passed away 3 days later.

## Discussion

Toxic leukoencephalopathy is a condition encompassing various non-specific neurological manifestations resulting from white matter lesions, due to the consumption of different toxic agents (chemotherapeutic drugs, immunosuppressants, environmental toxins, recreational drugs, etc.). We distinguish 2 forms: the most frequent one being the chronic form typically revealed by insidious manifestations, and the relatively rare and more severe one being the acute form.^1^^,^^2^ Toxic leukoencephalopathy induced by heroin consumption represents a rare entity, described only in its acute form, and that appears to affect almost exclusively the patients who have inhaled the drug.^3^

The inhalation of heroin vapours, also known as “chasing the dragon,” is a method of drug administration that dates back to the 1950s and is believed to have originated in China. The drug is typically placed on a piece of aluminium foil and heated from underneath using a match or a lighter. The powder transforms into a reddish-brown gelatinous substance, releasing white fumes that are inhaled using a straw or similar device.^3^^,^^4^ Many heroin users consider this method to be relatively safer than intravenous injections.^5^

The physiopathology underlying HLE and the reasons why it’s mainly caused by the inhalation of heroin vapours are poorly understood. First described in 1982 in a Dutch cohort, the clinical signs and their severity can vary, mild cases primarily present with cerebellar and bulbar involvement, including dysarthria and ataxia, accompanied by motor signs such as hyporeflexia, muscle weakness, and spasticity.^3^^,^^6^ More severe cases present with flaccid paralysis, sometimes accompanied by mutism, as observed in our patient.

The diagnosis should be suspected in a patient known to be a heroin user and presenting with acute neurological signs in the absence of other infectious or toxic causes mimicking similar symptoms. However, identifying this condition can sometimes be challenging due to difficulties in obtaining a detailed medical history in patients with severely impaired neurological abilities or due to the patient’s reluctance to disclose toxic substance abuse. Definitive diagnosis requires anatomopathological examination of brain samples, which is rarely performed in practice. Histologically, HLE is described as a “spongiform” leukoencephalopathy of the white matter, with the presence of vacuoles within oligodendrocytes and myelin sheaths, observed under electronic microscopy.^7^^,^^8^ Demyelination may be present but can be rather subtle; however, axons are generally spared.

Imaging plays a crucial role in HLE diagnosis, given the challenges in obtaining histological confirmation. MRI is the gold standard examination, and although there are no pathognomonic signs, the lesions pattern is often highly suggestive. Typical findings in toxic leukoencephalopathy including HLE consist of T2/FLAIR hyperintensities affecting both supratentorial and infratentorial white matter, associated with involvement to the corpus callosum. The lesions will usually show scattered hyperintensities on diffusion-weighted imaging and low ADC, This reflects the demyelinating nature of the lesions. Specific features include:^1^^,^^3^

- A vacuolated appearance of the lesions, representing the presence of fluid-filled cyst-like formations between nerve fibres, particularly well visualized on T2-weighted sequences as shown in Figure 1.
- The lesions tend to spare subcortical U fibres.
- Bilateral and symmetrical involvement of the posterior limbs of the internal capsule extending to the corticospinal tract.
- Butterfly-shaped symmetrical involvement of the middle cerebellar peduncles.
- Spectroscopy findings are non-specific and typically show a reduction in N-acetylaspartate and choline, as well as an increase in lactate levels.

The differential diagnosis is typically made with other toxic encephalopathies, but the presence of a history of heroin inhalation along with typical imaging findings (as described above) is usually sufficient to establish the diagnosis.

Management primarily focuses on symptomatic treatment, although supplementation with certain molecules such as coenzyme Q10, vitamin E, and vitamin C has shown some benefits in some patients. The prognosis varies depending on the severity of white matter involvement and clinical manifestations. In severe cases, mortality is very high, approaching 99%. In moderate cases, mortality is lower, but long-term neurological sequelae are common.^3^

## Learning points

- Although rare, heroin-induced leukoencephalopathy should be considered in polydrug users. A detailed medical history should be obtained to determine potential heroin consumption and the method of administration.
- MRI plays a crucial role in diagnosis due to the difficulty of histological confirmation.
- Radiological findings are not specific; however, a pattern of bilateral signal abnormalities sparing U fibres, middle cerebellar peduncles sign, involvement of the posterior limb of the internal capsule, together with a favourable clinical presentation, strongly suggests the diagnosis.

## Funding

None declared.

## Conflicts of interest

None declared.
